# Camptothecin in Cancer Therapy: Current Challenges and Emerging Strategies with Nanoemulsions

**DOI:** 10.3390/pharmaceutics17111414

**Published:** 2025-10-31

**Authors:** Heber Uriel Pérez-Ortega, Rubén Ricardo Córdova-Espíritu, Sebastian Cano-Serrano, Eduardo García-González, Micael Gerardo Bravo-Sánchez, Ma. del Carmen Orozco-Mosqueda, Hugo Jiménez-Islas, Gabriel Luna-Bárcenas, Francisco Villaseñor-Ortega

**Affiliations:** 1 División de Estudios de Posgrado e Investigación (DEPI), Tecnológico Nacional de México (TecNM), Instituto Tecnológico de Celaya (ITC), Celaya 38010, Guanajuato, Mexico; d2403038@itcelaya.edu.mx (H.U.P.-O.);; 2 Departamento de Ingeniería Bioquímica y Ambiental, Tecnológico Nacional de México (TecNM), Instituto Tecnológico de Celaya (ITC), Celaya 38010, Guanajuato, Mexico; d2103017@itcelaya.edu.mx (R.R.C.-E.); gerardo.bravo@itcelaya.edu.mx (M.G.B.-S.); carmen.orozco@itcelaya.edu.mx (M.d.C.O.-M.); hugo.jimenez@itcelaya.edu.mx (H.J.-I.); 3 Tecnológico Nacional de México (TecNM), Instituto Tecnológico de Roque (ITR), Celaya 38110, Guanajuato, Mexico; 4 Programa de Ingeniería en Biotecnología, Campus Celaya-Salvatierra, Universidad de Guanajuato, Mutualismo #303, Col. La Suiza, Celaya 36060, Guanajuato, Mexico; 5 Tecnológico de Monterrey, Instituto de Materiales Avanzados para una Manufactura Sostenible, Epigmenio González 500 Fracc. San Pablo, Querétaro 76130, Querétaro, Mexico

**Keywords:** camptothecin (CPT), cancer, topoisomerase I (Topo I), intrinsic pathway, extrinsic pathway, apoptosis, caspases, nanoemulsions, SNEDDS

## Abstract

Camptothecin (CPT) is a natural alkaloid with potent antiproliferative activity, mediated by the inhibition of Topoisomerase I (Topo I), an essential enzyme for deoxyribonucleic acid (DNA) replication. However, its clinical application has been limited by low solubility and the instability of the lactone ring under physiological conditions, both of which decrease its efficacy. Semi-synthetic analogs such as irinotecan (CPT-11) and topotecan (TPT) have been developed and approved for the treatment of various types of cancer; however, challenges related to drug resistance and side effects continue to arise. Therefore, nanomedicine and nanoparticle-based delivery systems, including nanoemulsions, liposomes, and antibody–drug conjugates (ADCs), emerge as promising strategies to improve the stability, bioavailability, and effectiveness of CPT, despite significant challenges such as scalability, pharmacokinetic variability, and regulatory requirements. This review discusses recent advances in CPT, its analogs, and these delivery platforms, highlighting its potential to optimize cancer therapy and reduce toxicity while outlining translational challenges such as scalability, pharmacokinetic variability, and regulatory requirements.

## 1. Introduction

The World Health Organization (WHO) describes cancer as a group of diseases that can occur in any organ or body tissue. This disease is marked by the uncontrolled growth of damaged cells that divide, grow, and spread uncontrollably, invading other tissues [1]. The formation of solid tumors involves six specific capabilities: sustained proliferative signaling, evasion of growth suppressors, disabling replicative mortality, induction of angiogenesis, cancer invasion or metastasis, and resistance to apoptosis or cell death [2].

Cancer represents one of the greatest health, social, and economic challenges of the 21st century. According to estimates from the International Agency for Research on Cancer (GLOBOCAN), nearly 20 million new cases and 9.7 million deaths attributable to this disease were recorded in 2022 [3]. Lung cancer remained the leading cause of cancer-related death, with around 1.8 million deaths (18.7%), followed by colorectal cancers (9.3%), liver cancer (7.8%), breast cancer (6.9%), and stomach cancer (6.8%) [4]. Given this situation, it is essential to seek more effective treatments that utilize anticancer agents with high antiproliferative activity and minimal side effects. Fortunately, nature offers a rich diversity of natural products that can be harnessed for the development of new drugs in the fight against this disease [5].

More than 65% of the medications used in cancer treatment are derived from plants and their derivatives. These compounds have been essential in the production of antiproliferative agents such as vincristine, vinblastine, doxorubicin, paclitaxel, among others, which have had a profound impact on the lives of countless cancer patients [6,7]. In 1966, Wall and Wani discovered camptothecin (CPT), initially extracted from the bark of *Camptotheca acuminata*, a plant species in the Camptotheca genus and Nyssaceae family, native to southern China [8]. *Camptotheca acuminata* is known for its fast growth in temperate climates. However, it does not tolerate cold well and requires a humid environment and fertile soil to flourish. CPT can be obtained from the fruit, roots, bark, branches, and leaves. However, in 1985, this compound was identified as an inhibitor of the enzyme topoisomerase I, an enzyme involved in the DNA replication process in eukaryotic cells [9]. It shows a wide range of antiproliferative activity, with more potent effects on colorectal, ovarian, breast, lung, and renal cancers, including those resistant to chemotherapy [10].

By the late 1990s, improved understanding of camptothecin (CPT)’s mechanism of action, chemistry, and pharmacology led to the development of analogs with better properties for clinical use [11], making it the second most important antiproliferative medicinal plant in the world, after *Taxus baccata* [12]. Additionally, it has been found that camptothecin and its derivatives exhibit other pharmacological effects, including anti-obesity, antiviral, and antifungal activities [13]. The global interest in camptothecin (CPT) and its derivatives has steadily increased over the past few decades, primarily driven by their clinical significance. Nearly twenty years ago, combined sales of CPT-11 and TPT already surpassed $750 million, setting a record in the oncology market [14]. Since 2018, this trend has sharpened, establishing CPT and its analogs as highly significant therapeutic and clinical agents worldwide [15].

Currently, commercial numbers indicate a strong market, supported by active trade of exports and imports between countries such as India, the USA, China, and Taiwan. This reflects the increasing international demand for camptothecin and its derivatives. However, its production remains a significant challenge, as roughly 1000 to 1500 tons of plant material are needed to produce just one ton of purified CPT. Globally, annual sales of CPT and related compounds are estimated at nearly $1 billion, confirming its significance as a key component of the current oncology pharmaceutical market [16].

In this review, we surveyed the literature in PubMed/MEDLINE, Scopus, and Web of Science (covering from 1966 to 2025; focusing on 2002 to 2025) using terms related to camptothecin, its clinical analogs (irinotecan/CPT-11, SN-38, topotecan), topoisomerase I inhibition, and delivery platforms (nanoemulsions, SNEDDS, lipid systems), including antibody–drug conjugates with CPT-type payloads. Using the keyword “camptothecin,” the initial results yielded 19,615 records in PubMed, 22,487 in Scopus, and 10,938 in Web of Science (totaling 53,040). Records from all databases were exported and de-duplicated in Mendeley (via DOI/title/year matching) to create a consolidated set. Screening was conducted in stages: by year (2002–2025), document type (original research, reviews, clinical studies), topical relevance to oncology/Topo I, formulation/delivery focus, and language/full-text availability, followed by full-text assessment against predefined inclusion/exclusion criteria. Citation counts were also used to prioritize influential and widely referenced studies. This process ultimately resulted in 224 studies that served as references.

## 2. Chemical Structure and Physicochemical Properties

**Figure 1:**
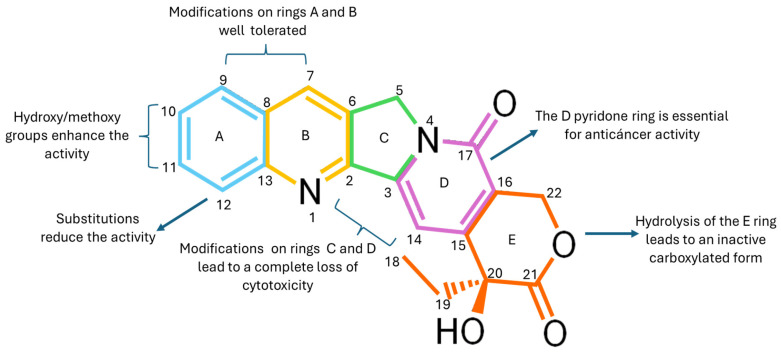
Chemical structure of CPT, highlighting the quinoline rings (A), conjugated (B and C), pyridone (D), and lactone (E), as well as key functional groups such as the hydroxyl (-OH). These structural regions are critical for its interaction with Topo I, and modifications introduced at specific positions, such as rings A, B, and E, have improved critical pharmacological properties, including solubility and stability, thereby enhancing its development as a basis for clinically useful analogs.

CPT is an alkaloid characterized by a planar pentacyclic structure composed of five fused rings. Its structure includes a pyrrole-(3,4-β)-quinoline system corresponding to rings A, B, and C, attached to a pyridone ring (ring D) as illustrated in Figure 1. Additionally, it has a chiral center at position C20 in the α-hydroxylactone ring (ring E) [17]. One of the main obstacles to the use of CPT is its low solubility and stability in water, as its structure is predominantly hydrophobic, which prevents its dissolution in this medium [18,19]. Although the molecule contains some polar functional groups, such as the lactone group, these are not sufficient to counteract the large hydrophobic surface of the molecule [17]. Furthermore, CPT has a partition coefficient (Log P) ranging from 1.30 to 2.60, indicating that it has a highly lipophilic solubility. This property enables it to cross the cell membrane [20,21].

To optimize its solubility without affecting its biological activity, modifications can be made to rings A, B, and C. However, rings D and E must remain practically intact, as they play a fundamental role in inhibiting Topo I, a key mechanism in the antiproliferative activity of CPT [22]. In an acidic environment, the E ring remains closed, allowing it to retain its efficacy [23]. When the pH exceeds 5.5, the lactone ring opens through a hydrolysis process, transforming into its carboxylate form, which results in a 90% reduction in its biological activity [24,25]. The half-life (t1/2) of the conversion of the lactone ring into its carboxylate form is 11 min, and only 5.3% of the total camptothecin remains in the lactone form, rendering it ineffective against cancer [26,27].

This pH-related behavior also affects its interaction with plasma proteins after administration. In the bloodstream, camptothecin binds to transport proteins such as albumin and alpha-1-acid glycoprotein (AAG), facilitating its distribution throughout the system [28,29,30]. At a pH of 5.5 or higher, the non-enzymatic hydrolysis of the lactone ring occurs rapidly and reversibly, generating an open-ring hydroxyl carboxylic acid. Human serum albumin has a higher affinity for the carboxylate form, favoring its conversion and further decreasing the amount of CPT in its active form [26,31], which limits its bioavailability and therapeutic efficacy, posing an obstacle to its clinical use [32,33]. Unfavorable pharmacokinetic conditions compromise biological activity, requiring higher doses or continuous applications to achieve a therapeutic effect [34]. To maximize efficacy and minimize side effects, some analogs have been developed by modifying the CPT structure [35,36].

## 3. Mechanism of Action

**Figure 2:**
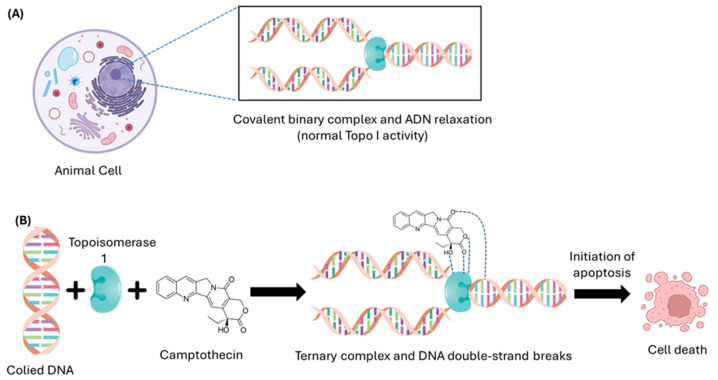
(**A**) Under normal conditions, Topo I transiently cuts a strand of DNA to relax the supercoiling generated at the replication fork and then re-ligates it, allowing normal replication to proceed. (**B**) In the presence of CPT, the drug binds to DNA and Topo I, forming a ternary complex that prevents re-ligation and re-coiling of the DNA strand, leading to the accumulation of breaks in the chain and thence causing cell death.

CPT exerts its antiproliferative activity by directly inhibiting Topo I, an essential enzyme in DNA replication, transcription, and repair processes [37,38]. Topo I modulates DNA supercoiling by generating transient single-strand breaks, allowing torsional relaxation through controlled rotation of the strand and subsequent re-ligation [39,40,41]. CPT, through its lactone ring, specifically binds to the Topo I–DNA complex via hydrogen bonds with amino acid residues of the enzyme, such as Arg364, Asn722, and Asp533, stabilizing this complex and blocking DNA re-ligation [39,40]. Although Topo I functions at a remarkably high speed (up to 6000 cycles per minute), it is highly susceptible to inhibition by agents like CPT [42]. In this way, CPT converts the standard catalytic action of Topo I into a stable complex that interferes with the progression of the replication fork, causing lethal DNA breaks and leading to apoptosis in tumor cells. Figure 2 schematically illustrates how CPT binds to the Topo I–DNA complex, inducing programmed cell death in cancer cells.

This inhibition converts the regular catalytic activity of Topo I into a stable Topo I–DNA–CPT complex, which interferes with the progression of the replication fork, causing lethal DNA breaks and leading to genotoxic stress, especially in cancer cells during the S phase [43]. The accumulation of these covalent Topo I–DNA–CPT complexes is cytotoxic and triggers apoptosis, a type of programmed cell death that primarily occurs through the mitochondrial intrinsic pathway, activated by DNA damage [44,45].

The intrinsic pathway is activated in response to DNA damage caused by CPT [46,47]. The p53 gene, known as the ‘guardian of the genome,’ detects this damage and coordinates the cellular response, promoting apoptosis [48]. Activation of p53 induces the transcription of proapoptotic proteins such as Puma, Noxa, and Bad, which antagonize antiapoptotic proteins of the BCL-2 family (BCL-XL, MCL-1, BFL-1, BCL-W, and BCL2L10) located in the mitochondria [49]. This allows the activation of Bax and Bak, responsible for forming pores in the mitochondrial membrane (VDAC), facilitating the release of proapoptotic proteins such as cytochrome c and Smac/DIABLO into the cytosol [50,51,52].

In the cytosol, cytochrome c, released because of mitochondrial permeabilization induced by CPT, binds to Apaf-1, forming the apoptosome that recruits and activates caspase-9, while RAIDD facilitates the activation of caspase-2 [53,54,55]. Subsequently, caspase-9 activates caspase-3, which in turn triggers the activation of caspase-activated DNase (CAD). This enzyme fragments DNA into segments of 180–200 base pairs and degrades structural proteins such as actin and nuclear lamins. This process ensures the controlled disintegration of the cell, while still maintaining the integrity of the cytoskeleton and nuclear membrane [56,57,58,59]. Additionally, mitochondrial stress mediated by CPT promotes the release of endonuclease G (Endo G), which migrates to the nucleus and contributes to DNA fragmentation and the formation of apoptotic bodies that are later eliminated by phagocytosis, thus protecting neighboring cells from damage [60,61].

**Figure 3:**
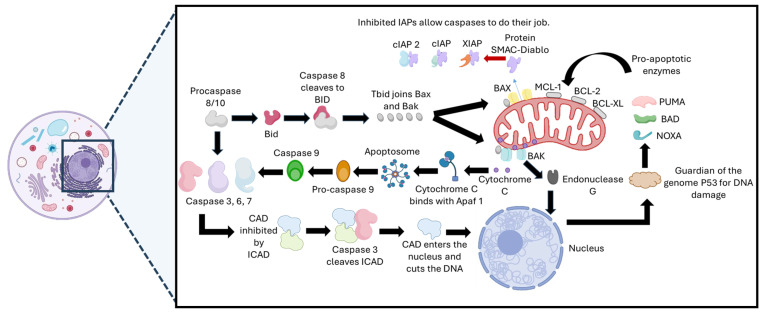
Activation of the intrinsic apoptosis pathway by CPT. The stabilization of the DNA–Topoisomerase I complex by CPT causes double-strand breaks during replication, which activates the p53-mediated DNA damage response. Activation of p53 regulates the expression of proapoptotic proteins of the Bcl-2 family, promoting mitochondrial permeabilization and the release of cytochrome c. This associates with Apaf-1 and procaspase-9 to form the apoptosome, which triggers the activation of effector caspases and ultimately leads to cell apoptosis.

In parallel, the apoptosis-inducing factor (AIF) is also released in response to CPT exposure, causing chromatin condensation and fragmentation independently of caspases, ensuring complete cell death [62]. Figure 3 schematically illustrates the intrinsic apoptosis pathway triggered by CPT.

The extrinsic pathway of apoptosis is activated by pro-apoptotic signals from the extracellular environment that interact with death receptors on the cell membrane, such as Fas (CD95) and TNFR1/TNFR2 [63,64]. However, CPT primarily exerts its effect through the mitochondrial intrinsic pathway by causing DNA damage. Activation of death receptors can reinforce CPT-induced apoptosis, but the drug does not need to exert its effect. When FasL or TNF-α ligands bind to their receptors, receptor trimerization occurs, leading to the recruitment of the adaptor protein FADD (Fas-Associated Death Domain), forming the death signaling complex (DISC) [65,66,67]. FADD, through its death effector domain (DED), recruits initiator procaspases such as procaspase-8 and procaspase-10, allowing their activation via autoproteolysis [68,69,70,71,72]. Activated caspase-8 initiates the apoptotic cascade by cleaving and directly activating effector caspases such as caspase-3 and caspase-7, thereby executing apoptosis through DNA fragmentation and cellular protein degradation [73,74,75]. Additionally, FADD associates with TRADD in TNFR1 signaling, promoting caspase-8 activation [76,77]. The protein Bid acts as a critical bridge between the extrinsic and intrinsic pathways, allowing signals initiated by death receptors to reinforce CPT-induced apoptosis. Caspase-8 cleaves and activates Bid, producing tBid, which translocates to the mitochondria and promotes outer mitochondrial membrane permeabilization. This action induces the release of cytochrome c and activates Bax and Bak, converging with the intrinsic pathway to amplify apoptosis [78,79,80]. In this way, CPT’s action, which primarily initiates the intrinsic pathway, is enhanced by integration with the extrinsic pathway, intensifying programmed cell death.

**Figure 4:**
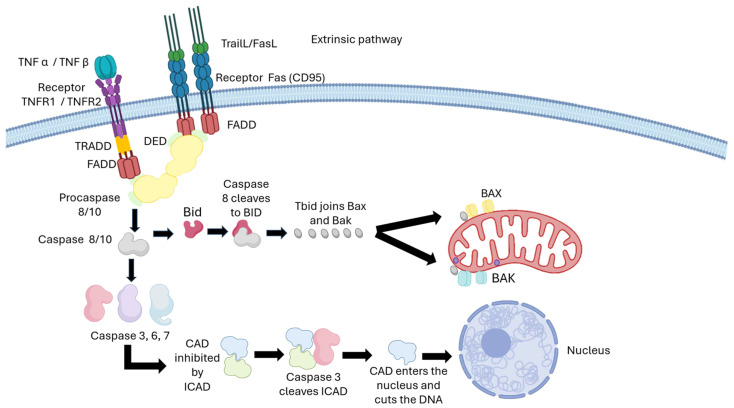
Extrinsic pathway of apoptosis and points of convergence with the mechanism of action of CPT. The activation of death receptors (TNFR, Fas/CD95, TRAIL) recruits adaptor complexes (TRADD, FADD) that activate caspases-8/10, initiating a signaling cascade that leads to the activation of effector caspases (3, 6, 7). Additionally, caspase-8 can cleave Bid, generating tBid, which activates Bax/Bak in the mitochondria and connects with the intrinsic pathway.

Figure 4 schematically illustrates the extrinsic pathway mediated by death receptors and its integration with the intrinsic pathway.

In mammals, caspases 2, 3, 6, 7, 8, 9, and 10 have been identified as key players in the apoptotic process [81]. These cysteine proteases, which are specific to aspartate, are expressed as proenzymes composed of an N-terminal domain, a large subunit (~20 kDa), and a small subunit (~10 kDa). These proenzymes are evolutionarily conserved and present in most cells [82].

**Figure 5:**
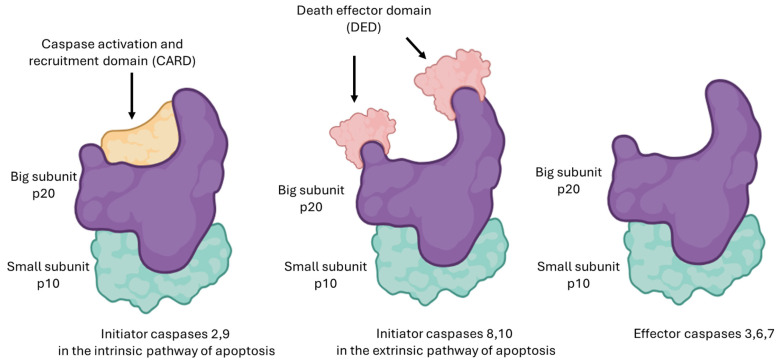
Structure of executioner and initiator caspases. All caspases contain a large subunit (p20) and a small subunit (p10) that form the catalytic site. Executioner caspases lack additional domains, while initiator caspases have regulatory regions such as DED or CARD, which allow their recruitment to activation complexes (DISC or apoptosome).

During apoptosis induced by CPT, DNA damage and the release of cytochrome c from mitochondria sequentially activate caspases, which are divided into initiator and effector caspases based on the protein interaction domains at their N-terminal end [83]. Caspases 8 and 10 contain a death effector domain (DED). In contrast, caspases 2 and 9 have a caspase recruitment domain (CARD), which facilitates their activation through dimerization or assembly into complexes such as the apoptosome, formed after CPT action. Caspase-9 acts as the initiator in the intrinsic pathway, while caspase-8 can amplify apoptosis by cleaving Bid to generate tBid, enhancing mitochondrial permeabilization. Finally, the effector caspases 3, 6, and 7 execute cell death by degrading essential structural and regulatory proteins [84,85]. Figure 5 shows the structure and function of initiator and effector caspases in CPT-mediated apoptosis.

DNA fragmentation, a characteristic stage of the final phase of camptothecin (CPT)-induced apoptosis, is mediated by caspase-activated DNase (CAD). Under normal conditions, CAD remains inactive by being bound to its inhibitor ICAD (Inhibitor of Caspase-Activated DNase) [86]. During apoptosis, the activation of caspase-3, as a result of the cascade initiated by CPT, leads to the cleavage of ICAD, releasing CAD and allowing controlled DNA fragmentation into fragments of approximately 180–200 base pairs [87,88].

**Figure 6:**
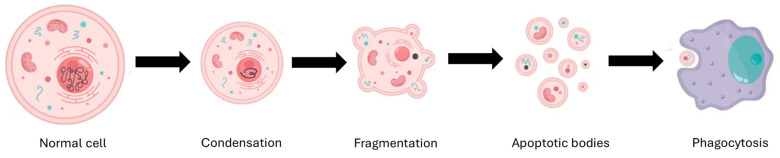
Apoptosis stages. The cell undergoes a series of changes, transitioning from a normal state to chromatin condensation and contraction. This is followed by nuclear fragmentation and the formation of apoptotic bodies, which are eliminated without triggering an inflammatory response.

Apoptosis results in a reduction in cell size, the formation of blisters in the membrane, chromatin condensation, DNA fragmentation, and the disassembly of cells into apoptotic bodies surrounded by a membrane [89]. Both apoptotic cells and apoptotic bodies are eliminated through phagocytosis by macrophages and non-specialized phagocytes, such as epithelial cells [90]. Figure 6 illustrates the process of apoptosis execution and elimination of apoptotic bodies.

## 4. Structural Changes in Camptothecin

### 4.1. Modifications on Rings A and B

**Figure 7:**
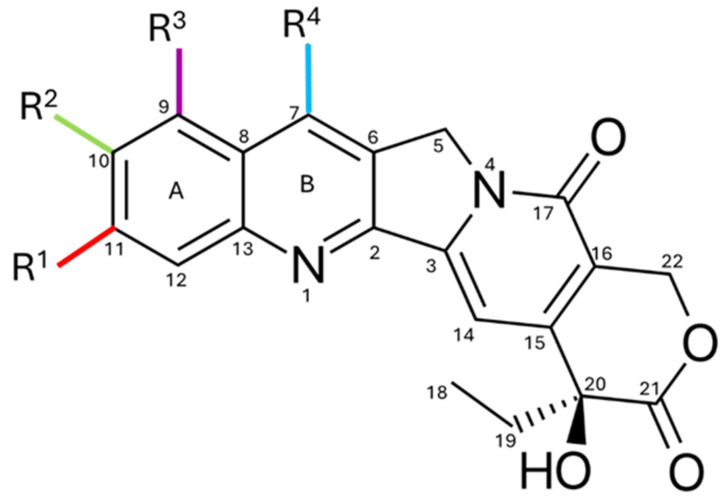
CPT structure with sites of structural modification. Substitutions on rings A and B, particularly, increase water solubility and antiproliferative potency, as seen in clinical derivatives such as topotecan and irinotecan.

Figure 7 shows the substitutions on the A and B rings of CPT. These modifications include the addition of amino and/or hydrophilic hydroxyl groups to improve solubility and potency [91]. Substitution at positions C7, C9, C10, and C11 enhances anticancer activity without compromising cytotoxicity [92]. CPT-11 and TPT are examples of semi-synthetic CPT derivatives that exhibit better water solubility due to structural modifications at position C9, thereby increasing their solubility under physiological pH conditions [93].

Studies also show that alkyl groups, such as ethyl or chloromethyl at C7, boost antitumor activity. This effect is linked to the favorable lipophilic interaction between these alkyl groups and the Topo I-DNA complex [92]. Extending the carbon chain length at C7 increases the molecule’s lipid solubility and is directly related to higher potency and greater stability of the E ring in plasma [94]. Substituents like amines, nitro groups, hydroxyl, chlorine, or bromine on ring A enhance the antitumor effectiveness of camptothecin, as seen with rubitecan, a 9-nitrocamptothecin analog with increased water solubility [92].

The molecular weight of CPT and its derivatives is approximately 400 Da, such as the 10-hydrocamptothecin (10-HCPT) analog, which has a substitution at the C10 position with a hydroxyl group (OH), substantially reducing the tendency for the lactone ring to open and increasing solubility [95], as well as the 7-ethyl-10-hydrocamptothecin (SN-38) analog, which has a hydroxyl group at the C10 position and an ethyl group at C7. Other analogs have been developed through modifications on rings A and B, such as lurtotecan (GG211) and exatecan (DX-8951f) [96].

The introduction of O-substituted oxime substituents at C7 of the B ring has led to the development of other potent compounds, such as gimatecan (ST1481) and namitecan (ST-1968), which are now in clinical studies [94,97].

### 4.2. Modifications on Rings C and D

As mentioned earlier, modifying the C and D rings of camptothecin results in a loss of activity because they are in the central region of the molecule and cause a decrease in its structural planarity [93]. It has been observed that substitutions at the C5 and C14 positions reduce the amide group and the carbonyl ester, making the molecule inactive. This is because the pyridone group and the carbonyl are crucial for stabilizing the ternary complex of Topo I, DNA, and CPT [98,99].

**Figure 8:**
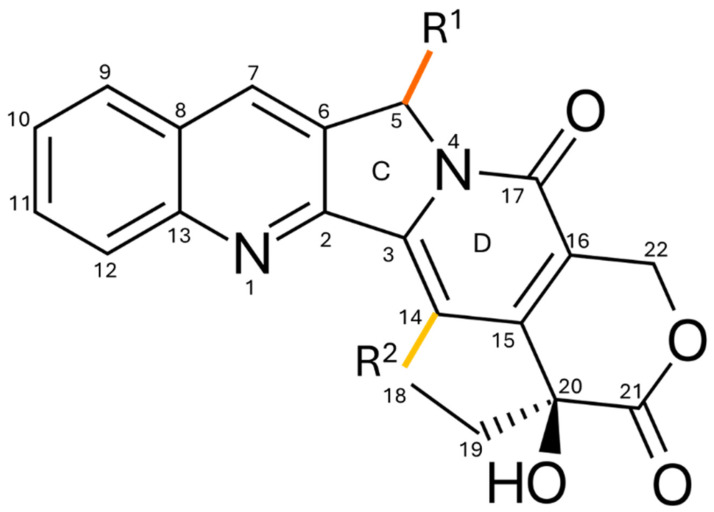
The C ring helps stabilize the planar conformation necessary for DNA intercalation without affecting the overall molecular planarity or its interaction with topoisomerase I (Topo I) and DNA. This structural feature has enabled the creation of clinical derivatives with better solubility and increased therapeutic effectiveness. In contrast, the D ring is part of the rigid core responsible for maintaining molecular planarity; any modification in this region tends to destabilize the interaction with Topo I and DNA, resulting in a significant loss of biological activity.

Modified CPT analogs have been developed on the C and D rings. However, none of them have been approved for clinical trials due to their low ability to inhibit topoisomerase I and their reduced antitumor activity [91]. Reports on substituents on the C/D rings are limited due to the scarcity of accessible carbons for modification and the difficulty of the synthetic routes required to obtain potential analogs. Figure 8 shows the carbons where substitutions can be made on the C and D rings in the CPT molecule.

### 4.3. Modifications on Ring E

The E ring of CPT is the most crucial part for inhibiting the enzyme Topo I. It binds to the enzyme through hydrogen bonds, so the E ring can only tolerate minimal modifications [100]. Various studies were conducted to replace the oxygen in the lactone ring with other atoms, such as nitrogen or sulfur. These efforts resulted in the synthesis of compounds like hydroxamic acid and 20-deoxifluorocamptothecin, which had reduced susceptibility to E ring opening, but their antiproliferative activity against cancer cells was compromised [91,101].

**Figure 9:**
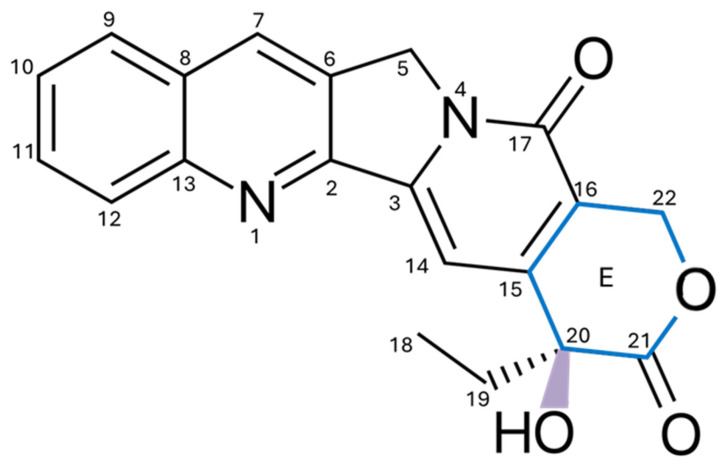
CPT structure highlighting the lactone ring (blue), which is essential for biological activity. The integrity of the ring is crucial for stabilizing the ternary complex with Topo I and DNA; its opening under physiological conditions renders the molecule inactive. This chemical sensitivity limits direct modifications to the E ring; therefore, derivatives aim to improve solubility and stability by modifying other regions of the molecule while preserving this critical ring.

However, Wall and colleagues demonstrated that the E ring of the molecule is essential in drug development. Therefore, they propose that the lactone ring remains intact. The activity of these compounds was reduced due to the loss of a hydrogen bond between the C20 group and the OH in the carbonyl group of the E ring [102,103]. These results led to one of the most significant conclusions in the field: the six-membered ring must remain intact for anti-tumor activity to occur. Figure 9 shows various molecules with substitutions on the E ring.

Among all the derivatives of CPT, only CPT-11 and TPT have successfully finished Phase I and II trials and received FDA approval. They have demonstrated clinical effectiveness and reduced toxicity compared to the original molecule in therapies for colon, lung, and ovarian cancer [99].

## 5. FDA-Approved Camptothecin Analogs

In the late 1960s, preclinical trials with sodium CPT, a water-soluble derivative, were initiated. However, severe toxic effects were observed, including sterile hemorrhagic cystitis, leukopenia, thrombocytopenia, and gastrointestinal toxicity, leading to the suspension of clinical trials with sodium CPT in the 1970s [104]. Over the next 20 years, researchers studied the drug in the laboratory, developing synthetic and semi-synthetic analogs of CPT aimed at improving its anticancer efficacy and safety [104,105].

### 5.1. Topotecan

**Figure 10:**
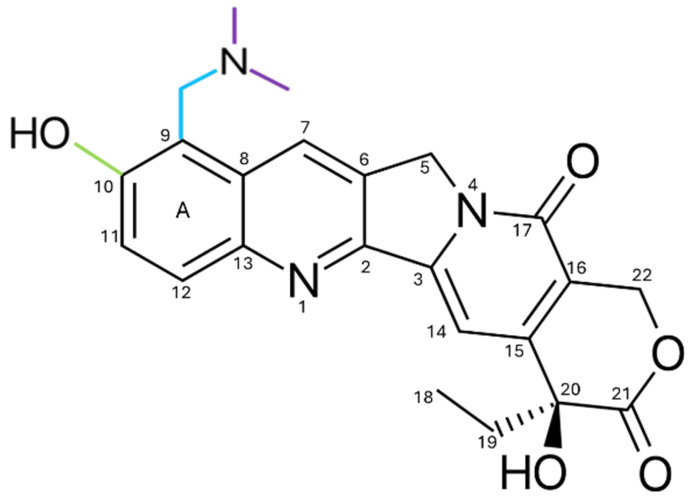
Structure of the TPT, derived from CPT. The introduced modifications are highlighted in colors, consisting of a dimethylaminomethyl group (blue and purple) and an additional hydroxyl group (green). These substitutions increase water solubility, improving formulation and clinical bioavailability, without altering the integrity of ring E, which is essential for interaction with Topo I and DNA.

Topotecan (TPT) is a hydrophilic CPT analog approved by the FDA in 1996 [106] for second-line treatment of metastatic ovarian cancer [107]. TPT incorporates a basic side chain at the C9 position of the A ring of 10-hydroxycamptothecin, as illustrated in Figure 10. The basic side chain contains amines that can accept protons (H^+^), forming ammonium ions. These ions can interact with negative ions (such as chlorides or sulfates) to form ammonium salts, thereby improving water solubility compared to CPT [93].

The CPT analogs are specifically targeted at Topo I [108]. Cancer cells are destroyed by damaging both ribonucleic acid (RNA) and DNA, which are essential components for cell division and proliferation [109]. In the USA, TPT is used for metastatic ovarian, cervical, and small-cell lung cancer (SCLC) [110]. Additionally, it is administered orally in capsule form or intravenously [111].

Pharmacokinetic analysis showed that the biological half-life of TPT is approximately 2–3 h. Doctors have recommended administering TPT at 1.5 mg/m^2^ of body surface area intravenously daily for 30 min over 5 days, with a 3-week interval between the start of each cycle [112]. As side effects, it usually causes severe neutropenia, as well as acute and delayed diarrhea [113]. Excretion primarily occurs through the renal and fecal routes. About 20% is recovered as the original drug (total TPT) in the urine, and 33% of the oral dose has been identified unchanged (total TPT) in the feces [114].

TPT remains a key treatment option for patients with Small Cell Lung Cancer (SCLC) who relapse or do not respond to initial chemotherapy and are not suitable for reinduction. A recent retrospective study involving 44 patients treated from 2015 to 2022 reported a median progression-free survival (mPFS) of 1.9 months and a median overall survival (mOS) of 5.6 months. By stratifying patients based on critical prognostic markers, including the Eastern Cooperative Oncology Group (ECOG-PS) scale, the presence of active brain metastases, and the systemic inflammation index (SII), researchers identified those most likely to benefit, achieving a median progression-free survival (mPFS) of 5.7 months [115]. This study provides current data on the clinical performance of approved CPT analogs, aiding in improving their effectiveness in clinical practice. The side effects of TPT may resolve after stopping treatment, making it easier to manage compared to drugs that cause permanent damage. Its primary toxicity does not significantly impact the blood system, unlike many chemotherapeutic agents that lead to anemia or severe immunosuppression [116].

### 5.2. Irinotecan

Irinotecan, also known as CPT-11, has been a crucial component in the treatment of solid tumors worldwide. This derivative remains a key component in combination therapies such as FOLFIRI (folinic acid, fluorouracil, and irinotecan) and FOLFIRINOX (leucovorin, fluorouracil, irinotecan, and oxaliplatin), which are types of combination chemotherapy used to treat pancreatic cancer that has spread to other parts of the body or is inoperable due to vascular involvement [116,117,118]. These therapies have proven effective in managing metastatic or advanced tumors, including colon, gastric, and pancreatic cancers [119].

**Figure 11:**
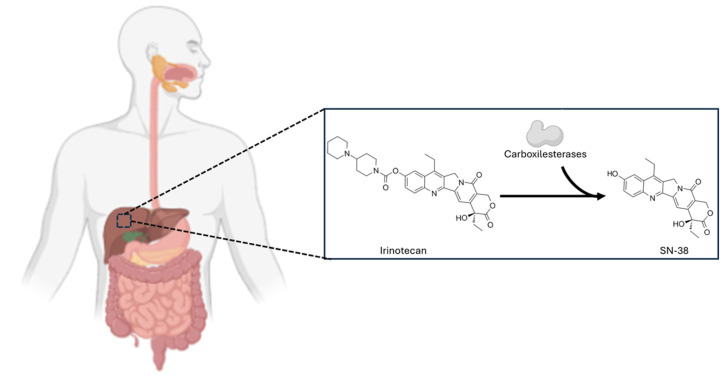
Enzymatic conversion of irinotecan to SN-38. The prodrug irinotecan is primarily hydrolyzed by carboxylesterases (CES), which remove the carbamate group and produce the active metabolite SN-CI.

CPT-11 is a water-soluble prodrug, which means it needs to be transformed within the body to become active. Due to its basic side chain, activation occurs through hydrolysis by hepatic carboxylesterases, releasing SN-38, the true cytotoxic agent responsible for the therapeutic effect against cancer [120], as shown in Figure 11. With these modifications, it behaves as a hydrophilic compound with a large volume of distribution estimated at nearly 400 L/m^2^ at steady state [121]. Treatment with CPT-11 may consist of intravenous infusions of 30 or 90 min at 350 mg/m^2^ every three weeks. Common adverse effects include neutropenia, diarrhea, nausea, vomiting, alopecia, and fatigue [122].

The elimination of CPT-11 and SN-38 is a process that depends on cellular membrane transporters of the ABC (ATP-Binding Cassette) family; the primary transporters involved in this process are the multidrug resistance gene (MDR1), P-glycoprotein (P-gp), MRP1, MRP2, and BCRP, with the most relevant being the canalicular multispecific organic anion transporter (cMOAT), also known as MRP2 (Multidrug Resistance-associated Protein 2). These transporters are overexpressed in tumor cell lines and, under physiological conditions, are expressed in epithelial cells of normal tissues such as the intestine, kidney, and liver, where they mediate the elimination of CPT by actively expelling it out of the cells, facilitating its excretion via bile, urine, or the intestine [120,123,124,125]. Table 1 summarizes the main clinical trials that have evaluated combinations with CPT derivatives in different oncological contexts.

The data show that these therapeutic strategies have demonstrated signs of efficacy. Additionally, the combination was associated with considerable toxicity, highlighting hematologic and hepatic effects, which limit treatment tolerability. These results suggest that, although integrating tyrosine kinase inhibitors with TPT is a promising approach, further optimizations in patient selection, therapeutic regimen design, and toxicity management are still necessary to solidify their role in clinical practice.

## 6. Unapproved Camptothecin Analogs

Beyond topotecan and irinotecan, several camptothecin analogs have been developed to enhance solubility, stability, and therapeutic effectiveness. While some have shown promising preclinical or early clinical results, most remain unapproved due to toxicity, limited efficacy, or pharmacokinetic issues. The following sections briefly highlight notable examples, including belotecan, exatecan, lurtotecan, and rubitecan.

### 6.1. Belotecan

7-[2-(N-isopropylamine)ethyl]-(20*S*)-camptothecin or Belotecan (CKD-602) has emerged as a promising alternative to TPT, with studies indicating a favorable toxicity profile. In a randomized phase II trial, CKD-602 was compared with TPT in 164 patients with relapsed, susceptible SCLC. The response rate (ORR) was found to be higher with CKD-602 (33%) compared with TPT (21%, *p* = 0.09). Furthermore, the median overall survival (OS) was significantly longer with CKD-602, at 13.2 months, compared to 8.2 months with TPT (HR = 0.69, 95% CI: 0.48–0.99). Although progression-free survival (PFS) did not show significant differences between the two groups, CKD-602 potentially offered a better tolerability profile. As second-line therapy for relapsed, refractory SCLC, the overall objective response (ORR) for CKD-602 was reported at 14%, with a PFS of 1.6 months, suggesting modest clinical benefits [129].

### 6.2. Exatecan

Exatecan mesylate (DX-8951f) is a water-soluble derivative of camptothecin, exhibiting greater potency and antitumor activity than other analogs. Currently, it is used as a payload in antibody–drug conjugates (ADCs), where the DXd variant binds to antibodies via tetrapeptide linkers. This compound exhibits high cytotoxicity, good cell permeability, and a bystander effect; however, its marked hydrophobicity makes direct conjugation with antibodies difficult, limiting its application. In phase I clinical trials, free formulations of DX-8951f showed dose-limiting toxicity, primarily severe myelosuppression, which reduced its potential as a standalone agent [130].

### 6.3. Lurtotecan

Lurtotecan (GG211) is a semi-synthetic derivative of CPT that stands out for its water solubility and potent antitumor activity. It has been primarily evaluated for the treatment of epithelial ovarian cancer. To optimize its efficacy and pharmacokinetic profile, GG211 has been formulated in unilamellar liposomes, resulting in prolonged plasma residence time in preclinical models and a 1500-fold increase in the area under the curve (AUC) compared to the free form. However, despite these advances, clinical trials did not show superior efficacy compared to other available analogs, and the appearance of neutropenia and thrombocytopenia limited its development. For these reasons, its clinical use has been limited, and it has not been adopted as a standard option in oncology [131].

### 6.4. Rubitecan

9-nitro-20(*S*)-camptothecin or Rubitecan (RFS-2000) is a semi-synthetic derivative of camptothecin. In preclinical studies, xenografts of human cancer implanted in nude mice, administered at maximum tolerated doses, showed complete inhibition of tumor growth in all 30 evaluated models, and total regression in 24 of them. These models included the most common types of cancer: lung, colon, breast, pancreas, ovary, prostate, stomach, melanoma, and leukemia. However, despite these encouraging results in the preclinical phase, early clinical trials (phase I/II) showed limited benefits against solid tumors, except for pancreatic cancer. Larger clinical studies are expected to clarify in the coming years whether rubitecan can establish itself as a therapeutic option in oncology [132].

**Figure 12:**
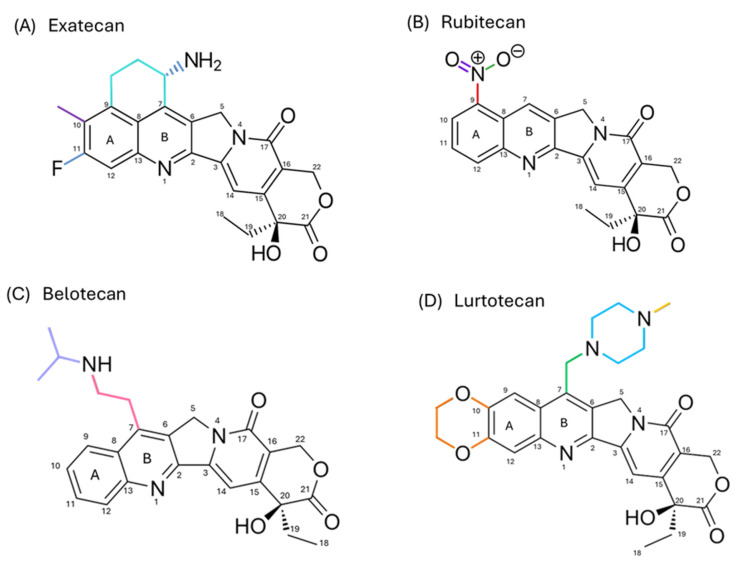
Representative chemical structures of semi-synthetic camptothecin analogs in clinical studies, with modifications only in rings A and B. (**A**) Exatecan contains structural modifications in both rings A and B. (**B**) Rubitecan displays alterations exclusively in ring B. (**C**) Belotecan also presents modifications confined to ring B. (**D**) Lurtotecan exhibits structural changes in both rings A and B.

Figure 12 shows the molecular structure of non-approved CPT analogs with modifications in rings A and B. These analogs have been evaluated in clinical studies and are still in the research phase.

Table 2 summarizes clinical trials of CPT derivatives evaluated (belotecan, exatecan, and rubitecan), which show improvements in tolerability and specificity. However, response inconsistency and toxicity persist, preventing their approval by the FDA. Therefore, safer formulations with approaches that maximize antitumor activity are needed.

In addition to their function as individual agents, they have demonstrated considerable benefit when used alongside other chemotherapeutic drugs. Their capacity to boost antitumor effects through synergistic mechanisms helps to achieve better clinical outcomes, slow down resistance, and reduce toxicity by allowing smaller doses of each drug.

## 7. Conjugates (ADC) Based on Camptothecin Derivatives

The main limitation of drugs used to eliminate cancer cells is that they also destroy normal cells. Researchers devised a plausible solution: combining these drugs with an antibody (conjugate). The drug remains inactive until the antibody enters the targeted cancer cell, at which point it is released inside the cell. The FDA in USA has approved thirteen ADCs, including two derivatives of camptothecin: trastuzumab deruxtecan (T-DXd, Enhertu^®^) and sacituzumab govitecan (Trodelvy^®^) [135]. These conjugates represent an innovative therapeutic strategy that combines three essential elements: a monoclonal antibody, a linker, and a cytotoxic agent. The monoclonal antibody functions as a vehicle, recognizing overexpressed antigens on cancer cells and minimizing damage to healthy tissues. Because of this specificity, the cytotoxic payload is released locally, which helps reduce systemic toxicity and improve the clinical safety profile [136].

IMMU-132, called sacituzumab govitecan (SG, Trodelvy^®^), is a third-generation ADC composed of a humanized monoclonal antibody directed against TROP2, a transmembrane glycoprotein overexpressed in various epithelial tumors. This antibody is conjugated, via a hydrolysable linker, to SN-38, the active metabolite of irinotecan (CPT-11) [137]. His therapeutic action is based on the inhibition of Topo I, which induces DNA damage and triggers selective apoptosis in tumor cells. In the clinical setting, SG has received FDA approval for handling patients with previously treated metastatic triple-negative breast cancer (TNBC), demonstrating superior efficacy and a more favorable safety profile compared to standard therapies [138].

Trastuzumab deruxtecan (T-DXd, DS-8201; Enhertu^®^) is an anti-HER2 ADC developed by Daiichi Sankyo^®^ in collaboration with AstraZeneca^®^. This conjugate combines the antibody trastuzumab with a derivative of CPT linked via a cleavable linker, ensuring efficient drug release inside HER2-positive tumor cells. T-DXd has demonstrated remarkable antitumor activity in patients with previously treated metastatic HER2-positive breast cancer, which led to its accelerated approval by the FDA in 2019 [139]. Currently, it is being evaluated in various clinical trials for HER2-positive gastric cancer, colorectal cancer with HER2 mutations or overexpression, and NSCLC with HER2 alterations, reporting promising preliminary results.

## 8. Resistance to Camptothecin

Cell death resistance has been identified as one of the key mechanisms in cancer progression, and its pathways are considered therapeutic targets [140]. Resistance to CPT and its analogs is a multifactorial phenomenon that limits their clinical efficacy. The main mechanisms include reduced intracellular accumulation of the drug, alterations in Topo I, and activation of DNA repair pathways [141]. In the case of TPT in cisplatin-resistant epithelial ovarian cancer, response rates are low (10–15%) due to decreased expression of Topo I and overexpression of ABC transporters such as ABCB1/P-gp and ABCG2/BCRP, which actively expel the drug and promote cross-resistance to multiple chemotherapeutic agents [142,143,144,145,146,147,148,149].

In the case of CPT-11, its effectiveness relies on its conversion to the active metabolite, SN-38, by carboxylesterase. The absence of conversion in the tumor decreases cytotoxicity, while selectively enhancing it may improve the response [150,151].

However, both irinotecan and SN-38 can be inactivated by UGT1A1, CYP3A4, and β-glucuronidase, which limits their clinical activity. In patients with colorectal cancer, a strong activation of NF-κB has been observed, which regulates pro-inflammatory factors and proto-oncogenes. Also, it induces the expression of PD-L1, thereby promoting immunosuppression and resistance to immunotherapy. Additionally, proteins such as osteopontin (OPN), survivin, and ISG15 are overexpressed, promoting proliferation, metastasis, and resistance. Silencing these proteins partially reverses resistance and increases sensitivity to SN-38 [152,153].

Genomic and transcriptomic studies have enabled precise mapping of the molecular basis of resistance. In human HAP1 cells exposed to TPT, in vitro evolution revealed that resistance is associated with a limited number of genomic variants. These variants were validated through RNAi and CRISPR-Cas9, confirming their functional role in modulating drug sensitivity [154]. It has been demonstrated that both mutations and overexpression of ABC transporters facilitate the expulsion of irinotecan and SN-38, reducing their efficacy. Additionally, transcriptomic networks mediated by OPN, NF-κB, PD-L1, survivin, and ISG15 reinforce cell survival and chemoresistance [153].

Resistance to CPT also involves mechanisms of DNA repair and degradation: repair of double-strand breaks mediated by BRCA1/2 and RAD51, along with the degradation of the trapped Topo I complex via ubiquitin/proteasome, reduces accumulated damage and compromises therapeutic efficacy. Overexpression of ABCG2/BCRP and other transporters, such as MRP1, is associated with lower intracellular CPT accumulation and poorer clinical response in multiple tumors, including breast, glioma, hepatocellular, and non-small cell lung cancers [155,156,157,158,159,160,161,162].

Among the most relevant mechanisms of clinical resistance are the constitutive activation of HIF, which promotes angiogenesis and tumor progression, generating resistance even against HIF-2α inhibitors; p53 dysfunction, which reduces apoptosis and facilitates resistance to multiple therapies; and the compensatory activation of the Akt/mTOR pathway, which has driven the development of next-generation inhibitors and combined strategies with tyrosine kinase inhibitors (TKI). Additionally, activation of the MEK/ERK and Wnt/β-catenin pathways has been linked to resistance to TKI, although experimental studies have demonstrated that inhibiting these pathways can overcome this resistance [163].

Resistance to CPT and its derivatives arises from the interaction between alterations in Topo I, overexpression of ABC transporters, activation of DNA repair mechanisms, and changes in cell signaling. This knowledge is essential for designing strategies to overcome it, including the development of transporter inhibitors, rational combinations with immunotherapy, and innovative formulations that optimize delivery and intracellular accumulation, thereby increasing therapeutic efficacy [164,165,166].

## 9. Management of Side Effects of Camptothecin

The adverse effects of CPT and its analogs include diarrhea, nausea, vomiting, neutropenia, and hemorrhagic cystitis [167]. Chemotherapy-induced diarrhea is typically treated with loperamide and atropine, which slow intestinal motility and improve patient comfort [168,169,170,171]. Hemorrhagic cystitis requires intravenous hydration, bladder irrigation, and, in severe cases, blood transfusion [172,173]. Antiemetics help prevent and control nausea and vomiting, although their effectiveness varies depending on individual response [174]. Neutropenia is prevented or treated with granulocyte colony-stimulating factors (G-CSF), especially in high-risk chemotherapy regimens [175,176].

Despite the potential of CPT for therapy, its effectiveness is limited by issues such as high toxicity, unstable lactone rings, and poor water solubility, which reduce its bioavailability in the bloodstream [177]. Consequently, developing a more effective delivery method is essential, and nanotechnology is among the most promising approaches.

## 10. Research and Current Advances

This section outlines the framework and sequence of topics: the biological basis for nanoscale systems interacting with membranes and organelles to locate cancer cells and control drug release; an overview of platforms (such as nanogels, polymeric nanoparticles—like micelles, liposomes, dendrimers—and inorganic materials) and the features that influence their performance; a detailed discussion of nanoemulsions—including formulation, characterization, and administration routes (oral and parenteral)—along with SNEDDS and related lipid systems; targeting strategies, comparing passive EPR-driven accumulation with ligand-mediated active uptake and stimulus-responsive release; and oncology applications, covering detection, imaging, and clinical experiences with CPT derivatives.

### 10.1. Nanotechnology in Medicine

Nanotechnology in the medical field is a rapidly growing area focused on detecting, treating, and preventing diseases using materials at the nanoscale. These systems, about the size of one thousand human cells and similar in dimension to biological molecules like enzymes and receptors, can interact directly with cell membranes and intracellular structures. Because of this ability, nanoparticles (NPs) can precisely locate disease sites and deliver therapeutic agents to targeted areas [178]. This technology has transformed the diagnosis and treatment of cancer, heart diseases, autoimmune disorders, and infections by surpassing biological barriers that traditional drugs cannot cross. As a result, it has led to the creation of more sensitive medical tools, highly accurate drug delivery systems, and personalized therapies [179]. These innovations improve treatment effectiveness by enhancing medication delivery directly to their intended targets [180]. Furthermore, NPs-based delivery systems have been engineered to enhance pharmacokinetic and pharmacodynamic profiles, increasing bioavailability, lowering systemic toxicity, and enabling a more controlled therapeutic response [181].

### 10.2. Types of Nanoparticles

Each type of nanotechnological platform has specific structural and functional features that determine its clinical potential. In the case of nanogels, their ability to gradually release the drug from the matrix allows for maintaining consistent plasma levels over long periods. This property, along with their controlled degradation, positions them as a suitable therapeutic strategy for chronic diseases that require the sustained, effective, and safe release of the active ingredient [182].

Polymeric nanoparticles (PNPs), including polymeric micelles, liposomes, and dendrimers, have proven to be effective systems for improving the solubility and stability of hydrophobic compounds, thereby helping to reduce systemic toxicity and enhance therapeutic efficacy. Recent advancements in polymer chemistry have enabled the creation of smart PNPs that can respond to specific physiological stimuli, such as pH changes, temperature shifts, or enzymatic activity, allowing for controlled and site-specific drug release [183].

Among inorganic nanomaterials, gold nanoparticles are notable for their relative biocompatibility and their ability to modulate optical properties, which allows them to be used in both imaging diagnostic techniques and photomedicine therapies. Additionally, functionalized nanotubes with antibodies that target tumor antigens enable highly selective release; when exposed to laser radiation, they absorb the energy and convert it into heat, destroying malignant cells without harming the surrounding healthy tissue [184].

Nanoparticle-based delivery systems (NPs) enable the encapsulation of hydrophobic drugs, shielding them from enzymatic degradation and extending their half-life in circulation. This advantage mainly stems from the enhanced permeability and retention (EPR) effect, which causes NPs to accumulate principally in tumor tissue due to the abnormal and highly permeable microvasculature typical of tumors. Consequently, the drug’s bioavailability is improved, and its therapeutic effectiveness is increased [185].

**Figure 13:**
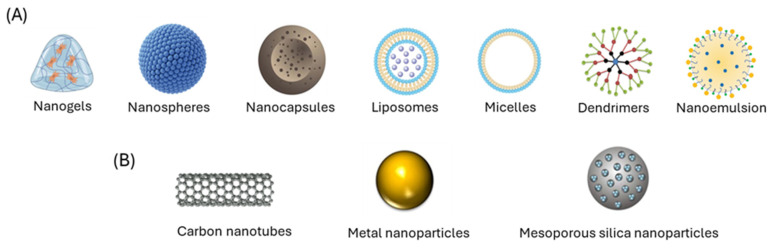
Main nanotechnology systems applied in drug delivery. These systems improve the solubility, bioavailability, and controlled release of therapeutic agents, offering advantages over conventional formulations. (**A**) All organic nanocarriers are included, such as dendrimers, micelles, polymeric nanoparticles, liposomes, polymersomes, drug–polymer conjugates, and protein-based nanoparticles. (**B**) All inorganic nanocarriers are included, such as carbon nanotubes, gold nanoparticles, and mesoporous silica nanoparticles.

There are different types of systems, such as nanoemulsions (NEs), polymeric nanoparticles (PNPs), liposomes, and others, characterized by particle sizes ranging from 1 to 200 nm [186,187]. Figure 13 shows several examples of drug delivery systems.

### 10.3. Formulation and Characterization of Nanoemulsions

**Figure 14:**
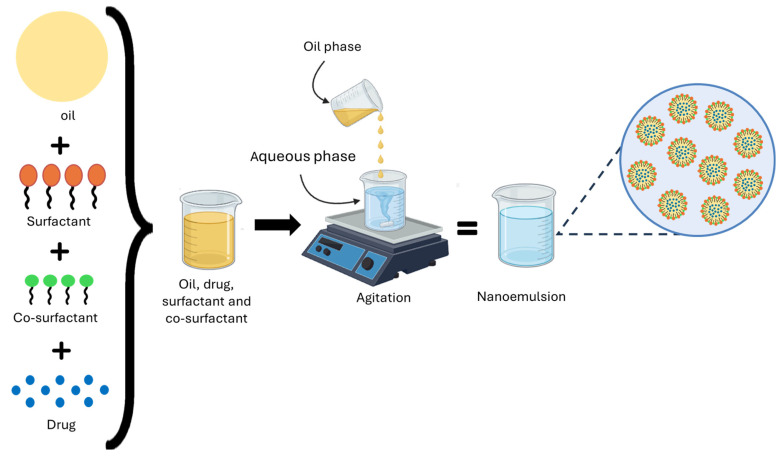
Self-nanoemulsifying drug delivery systems (SNEDDS) preparation. The lipophilic drug is mixed with oil, surfactants, and co-surfactants to form an isotropic preconcentrate. Upon dispersing this preconcentrate in water under gentle agitation, stable nanoemulsions are generated, which improve solubility and oral bioavailability.

These systems can achieve the desired therapeutic effect, a feature that current technologies lack [188]. Within this approach, NEs have emerged as an innovative strategy for drug delivery, due to their unique properties that enhance the solubility and bioavailability of hydrophobic active ingredients [189]. NEs consist of immiscible liquids, such as oil and water, which form a single phase through an emulsifier, like surfactants and co-surfactants [190]. NEs are lipid-based and serve as a promising tool for delivering and improving the bioavailability of hydrophobic drugs. Most drugs are hydrophobic (lipophilic) in nature, which causes low solubility and bioavailability issues [191]. These systems can be used for developing pharmaceutical formulations due to their small particle sizes, making them suitable for topical, ocular, and intravenous administration [192]. Figure 14 shows the NEs structure.

Lipid formulations are divided into four types. Type I formulations include only lipids, such as triglycerides and drugs. Type II includes oils and O/W soluble surfactants. Type III combines lipids with surfactants and co-solvents to further facilitate self-emulsification, thereby increasing efficiency during the digestion process. Finally, Type IV contains only water-soluble surfactants and co-solvents (no oils) [193]. Transmission electron microscopy (TEM), scanning electron microscopy (SEM), Fourier Transform Infrared Spectroscopy (FTIR), dynamic light scattering (DLS), and Zeta potential analysis are some of the most valuable and straightforward techniques used to study the shape, size, chemical composition, and surface charge of particles [194].

For this reason, NEs are expected to be administered orally. This approach is mainly used to enhance the solubility and bioavailability of lipophilic drugs, as the nanoemulsion protects the active ingredient and helps its absorption in the intestine. However, oral administration faces significant challenges, such as low solubility, limited permeability, rapid metabolism, and instability in the gastrointestinal environment. Despite these issues, the oral route remains the preferred method for patients due to additional benefits such as reducing the risk of infections, thrombosis, and complications related to catheters [195,196,197].

To overcome these barriers, various nanocarrier systems have been developed, such as liposomes, lipid nanoparticles, double emulsions, cyclodextrins, proteins, and polymers like chitosan [198]. These systems protect active ingredients from the hostile environment of the gastrointestinal tract and enzymatic degradation, increasing their stability in biological fluids and their bioavailability [199]. Lipid nanocarriers are promising because they allow the encapsulation of hydrophobic compounds, reduce adverse effects, and prolong systemic circulation [200]. Furthermore, the evolution toward next-generation smart NPs has opened the possibility of evading immune recognition and selectively directing drugs toward tumor cells or their receptors [201], thereby increasing their effectiveness when combined with other therapeutic strategies [202].

### 10.4. Targeted Delivery of Nanoemulsions

**Figure 15:**
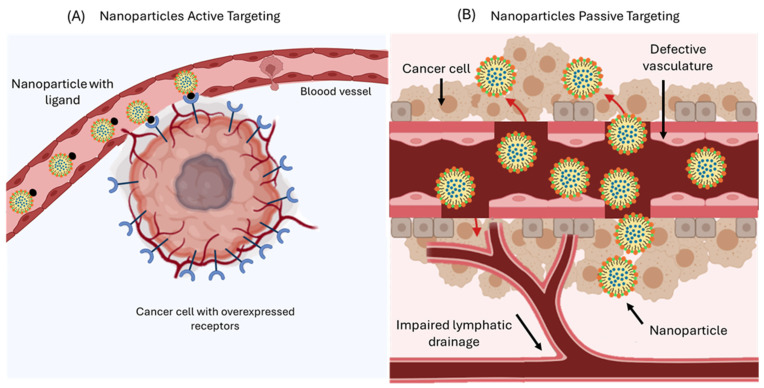
Schematic representation of targeted drug delivery using functionalized nanoparticles. (**A**) the effect of enhanced permeability and retention (EPR) is shown, through which nanoparticles passively accumulate in tumor tissue due to abnormal vascularization. (**B**) active targeting is illustrated, where nanoparticles are coated with specific ligands that recognize and bind to overexpressed receptors on the surface of tumor cells, facilitating their internalization and increasing therapeutic efficacy.

Drug delivery systems can be incorporated into cancer therapies, offering improvements in the pharmacokinetic parameters of therapeutic agents, reducing systemic toxicity to normal tissue, and minimizing adverse effects associated with conventional cancer treatments [203]. With this approach, nanoparticles can enter cancer cells through two distinct mechanisms, passive targeting and active targeting, as illustrated in Figure 15.

In passive targeting, these systems benefit from the characteristics of tumor tissues, such as the EPR effect, which allows NPs to concentrate in cancerous tissue [204]. Many tumors exhibit abnormal angiogenesis, resulting in permeable blood vessels with fenestrations and poor lymphatic drainage, characterized by elevated interstitial fluid pressure. This environment facilitates the penetration of NPs into the interstitial pores, which range in size from 100 to 780 nm [203]. NPs whose size typically ranges from 20 to 200 nm are suitable for taking advantage of this effect, as their size allows them to pass through these pores and accumulate in tumor tissue. Additionally, their retention is prolonged due to the low lymphatic drainage of the tumor. This targeted delivery can be further optimized through internal stimuli, such as acidic pH, specific enzymes, or alterations in redox potential, as well as external stimuli like temperature, magnetic fields, or radiation, thereby enhancing controlled drug release and reducing systemic side effects [205].

Active targeting exploits the biofunctionalization of NPs surfaces by using ligands with high affinity and specificity for receptors that are overexpressed on tumor cells [206]. This approach reduces systemic toxicity by avoiding healthy cells [207]. Controlled drug release should accumulate selectively and quantitatively at the therapeutic target, regardless of the route of administration [205].

Molecules such as folic acid, transferrin, biotin, and hyaluronic acid show a high affinity for receptors that are often overexpressed on tumor cells. Folic acid exhibits differences in its internalization pathway: in normal cells, it crosses the membrane via transmembrane transport, while in tumor cells, it enters through folate receptors that facilitate endocytosis. This basis has allowed the functionalization of the surface of nanoemulsions or other delivery systems with folic acid, thereby promoting selective internalization into cancer cells and intracellular drug release [208].

Nowadays, encapsulating active ingredients in NPs is a key strategy for directing drugs toward tumors or diseased organs, reducing damage to healthy tissues, and increasing therapeutic efficacy [209]. These nanoparticles can even penetrate the cell nucleus, release the active ingredient, and induce cell death [209,210].

### 10.5. Nanotechnology in the Oncology Field

Early diagnosis of cancer continues to be one of the main clinical challenges, as most cases are identified at advanced stages (III or IV), when the chances of curative treatment are limited. In response to this issue, nanotechnology has emerged as a promising tool to facilitate the early detection of tumors in different organs [211]. This discipline allows for the identification of tumor biomarkers in the extracellular microenvironment, as well as the application of in vivo bioimaging techniques [212].

NPs, because of their size, can cross cell membranes, making them excellent tools for detecting cancer cells and accurately locating affected areas [213]. These NPs can be engineered to specifically bind to receptors that are overexpressed on cancer cells, aiding their visualization and analysis. This method enhances the accuracy of early detection, tracking tumor progression, and reducing the time needed for clinical diagnosis [214].

The development of nanotechnology-based biosensors represents another prominent application in this field. These devices can identify specific biomarkers in blood or other bodily fluids, enabling the detection of molecular alterations associated with cancer in its early stages [215]. Nanotechnology aimed at cancer diagnosis offers a more precise, non-invasive, and efficient way to identify the disease. However, challenges related to standardization, sensitivity, and reproducibility of these methods persist, making it essential to continue optimizing nanotechnology-based diagnostic systems to improve prognosis and early detection of cancer and other pathologies in the future [212,214].

Table 3 presents the clinical trials conducted with NPs with CPT and their derivatives, designed to improve their stability, bioavailability, and therapeutic profile. These studies reflect advances in the use of nanostructured systems as a strategy to optimize drug release and reduce adverse effects associated with the free molecule.

CPT derivatives formulated in nanotechnology-based delivery systems demonstrate a superior toxicological profile compared to their non-encapsulated counterparts. As summarized in Table 1, Table 2 and Table 3, liposomal CPT-11, nanoparticle conjugates of CPT (CRLX101 and NLG207), and CPT-11-loaded nanomicelles (IH-NM) have shown a significant reduction in systemic and hematological toxicity, as well as improved overall tolerability and safety, even at higher doses. These findings suggest that nanostructured systems not only enhance the stability and bioavailability of the drug but also substantially mitigate adverse effects, representing a promising platform for safer and more effective clinical application of CPT derivatives.

Despite technological advances, NEs still face limitations that hinder their clinical use. The main challenges include the difficulty of ensuring scalable and reproducible production processes. Large-scale manufacturing requires specialized equipment and can lead to variations in particle size, increased costs, and waste. Additionally, uncertainties remain regarding their long-term safety, potential to trigger immune responses from specific components, and limited understanding of their pharmacokinetics and biodistribution, which can result in unpredictable therapeutic effects [220].

The regulatory environment still poses a significant challenge, as the lack of uniform guidelines and uncertainty regarding their long-term safety continue to delay approval by agencies such as the FDA [221]. Although lipid NE and polymer-based systems have demonstrated adequate biocompatibility and low toxicity in vivo models, a comprehensive chronic safety evaluation and the generation of solid clinical evidence in humans are still needed to support their incorporation into routine practice [222].

In the specific case of CPT, different systems have demonstrated improved solubility and enhanced antiproliferative activity; however, solid clinical evidence is still needed to support its implementation. Meanwhile, next-generation nanocarriers offer an emerging approach that should be explored in combination with immunotherapy and targeted therapies to optimize the clinical response [223]. Recently, countries such as the United States, the United Kingdom, Japan, and Germany have spearheaded the development of this technology due to their scientific advancements. Meanwhile, nations like Mexico, Colombia, and Brazil are still in the consolidation phase. Nonetheless, nanomedicine holds the potential to revolutionize the treatment of chronic and cancer-related diseases, which have negatively impacted the quality of life for millions over many decades [224].

## 11. Prospects

CPT derivatives remain essential anticancer agents, and advanced delivery methods such as nanoemulsions, liposomes, and ADCs improve their effectiveness and reduce toxicity. New techniques, such as reverse targeting and the use of anti-idiotypic distribution enhancers, combined with targeted therapies and immunotherapies, are being studied in clinical trials and could further enhance the effectiveness, selectivity, and clinical utility of CPTs.

Future research should optimize the nanoemulsion composition (droplet size, interfacial film, and zeta potential) and integrate drugs with immunotherapy to maximize tumor cytotoxicity and the antitumor immune response. However, the clinical translation of these platforms requires overcoming additional challenges, such as scaling up their production, harmonizing manufacturing protocols, obtaining regulatory approval under international standards, and assessing long-term toxicities to ensure solid safety profiles.

Together, these innovations establish CPTs and their advanced delivery platforms as novel support of modern oncology, offering more effective, safer, and more precise treatments that improve patients’ quality of life and accelerate their clinical implementation within a rigorous regulatory and translational framework.

## 12. Conclusions

CPT remains one of the most iconic molecules in the history of anticancer drug development, due to its highly specialized mechanism for targeting Topo I, which causes irreversible DNA damage and triggers cell apoptosis. Its clinical use has been limited by its chemical instability, low solubility, and systemic toxicity related to traditional administration. FDA-approved semi-synthetic analogs, such as CPT-11 and TPT, have made significant advances in efficacy and pharmacokinetics. However, they still face issues like acquired resistance, limited bioavailability, and treatment-related adverse effects.

The study of new structural modifications and conjugates, such as ADCs, offers promising alternatives to improve selectivity against cancer cells and expand the therapeutic range of CPT and its derivatives. Simultaneously, the incorporation of nanotechnological approaches, particularly nanoemulsions, has opened a new chapter in the development of CPT. These drug delivery systems protect the active form of the molecule, enhance its physicochemical stability, increase solubility in biological media, and direct its release toward tumor tissues through passive (EPR effect) or active (surface ligands) mechanisms. NEs not only provide an effective way to reduce toxicity and prolong plasma half-life but also facilitate co-administration of synergistic agents and treatment personalization. The rational combination of chemical synthesis of new derivatives and engineering of delivery systems based on nanoemulsions could mark the beginning of a new generation of anticancer formulations with greater efficacy, optimized safety, and significant clinical impact, thereby contributing to improved quality of life for patients. Advancing these strategies requires close multidisciplinary collaboration, precise regulatory guidelines, and solid clinical evidence to validate their therapeutic performance. This establishes CPT as a promising support in the evolution of modern oncological therapies and future precision medicine.

## Figures and Tables

**Table 1:** Relevant clinical trials of camptothecin derivatives.

Drug	Cancer Type	Clinical Phase	Result	Toxicity Profile	Ref.
Nal-IRI (nanoliposomal Irinotecan) vs. Topotecan	Small Cell Lung Cancer (SCLC) relapsed	Phase III, randomized	Similar OS and PFS; Nal-IRI showed higher ORR in some analyses; did not consistently achieve superiority	Nal-IRI: neutropenia, diarrhea (like irinotecan); topotecan: intense myelosuppression	[126]
BI354 (ADC: trastuzumab- CPT derivative)	HER2+ metastatic breast cancer and solid tumors	Phase I, global	ORR 58%; DCR 90.9%; DoR 12 m: 71.8%; PFS/OS not achieved; OS 9 m: 96.2%	Myelosuppression, anemia, nausea	[127]
Pazopanib + Topotecan	Platinum-resistant ovarian cancer	Phase II, randomized	Improved ORR in PP but with greater toxicity; pazopanib reduced bioavailability (−18.8%) but without impact on AUC; no PK-tox correlation	Dose-limiting toxicity in 23% (hematological and hepatic) in combination	[128]

OS: overall survival, PFS: progression-free survival, ORR: objective response rate, DCR: disease control rate, PK-tox: pharmacokinetics-toxicity correlation, DoR: duration of response, PP: Progression Parameters, AUC: Area Under the Curve (pharmacokinetic exposure).

**Table 2:** Summary of clinical trials of camptothecin derivatives.

Drug	Cancer Type	Clinical Phase	Result	Toxicity Profile	Ref.
Belotecan	Sensitive-relapsed small-cell lung cancer (SCLC)	Phase II	ORR 33%; DCR 85%; median OS 13.2 months; PFS not significantly different	Acceptable safety profile; higher treatment completion rate (53% vs. 35%); well tolerated in patients < 65 years	[133]
Exatecan	Solid tumors, including sarcomas	Phase I	Some antitumor activity: partial responses reported in solid tumors, including a patient with sarcoma	Neutropenia, Thrombocytopenia hematological, nausea, vomiting, diarrhea, elevated hepatic transaminases, asthenia, alopecia	[134]
Exatecan mesylate	Advanced soft tissue sarcomas (STS)	Phase II	CBR: 60% leiomyosarcoma; 53% non-leiomyosarcoma; 3-month PFS: 56% leiomyosarcoma; 26% non leiomyosarcoma; ORR 0%	Neutropenia, thrombocytopenia, anemia, dyspnea, fatigue	[134]
Rubitecan	Advanced soft tissue sarcomas (STS)	Phase I/II	Chordoma ORR 7%, mTTP 9.9 weeks. soft tissue sarcomas ORR 4%, mTTP 8.0 weeks; gastrointestinal stromal tumors mTTP 8.3 weeks	Anemia, hyperglycemia, nausea, leukopenia	[134]

OS: overall survival, PFS: progression-free survival, ORR: objective response rate, DCR: disease control rate, CBR: clinical benefit rate, mTTP: median Time To Progression.

**Table 3:** Summary of clinical trials with camptothecin NPs and derivatives.

Drug & Sponsor	Target	Clinical Trial	Cancer Type	Toxicity Profile	Ref.
NLG207 (Camptothecin nanoparticle–drug conjugate) + Enzalutamide *NewLink Genetics Corporation*	Topoisomerase I Androgen receptor inhibitor	Phase II	Metastatic castration-resistant prostate cancer (mCRPC) post-enzalutamide	Noninfectious cystitis (Grade 3) and myelosuppression; poor tolerability at 12 mg/m^2^	[216]
Liposomal Irinotecan *Ipsen* *Biopharmaceuticals* *Servier*	Topoisomerase I	Phase I	Metastatic breast cancer, including active brain metastases	Diarrhea (27.6%), nausea (17.2%), fatigue (13.8%), asthenia (10.3%), hypokalemia (10.3%); no treatment-related deaths	[217]
IH-NM (Irinotecan-loaded nanomicelles) *National Center for Nanoscience and* *Technology of China*	Topoisomerase I	Phase I	Colorectal cancer (CRC)	Improved tolerability (1.56× higher MTD vs. free CPT-11), reduced systemic toxicity, and no hematologic toxicity observed	[218]
CRLX101 (Camptothecin nanoparticle–drug conjugate) *Cerulean Pharma Inc.*	Topoisomerase I	Phase I/II	Locally advanced rectal cancer (with chemoradiotherapy)	Lymphopenia (1 Grade 4 case); overall excellent safety and feasibility	[219]

## Data Availability

Not applicable.

## Abbreviations

The following abbreviations are used in this manuscript:

10-HCPT	10-hydroxycamptothecin
AAG	Alpha-1-acid glycoprotein
ABC	ATP-binding cassette
ADC	Antibody–drug conjugate
AIF	Apoptosis-inducing factor
Akt	AKT serine/threonine kinase
Apaf-1	Apoptotic protease-activating factor 1
AUC	Area under the curve
Bak	BCL-2 homologous antagonist/killer (pro-apoptotic effector)
Bax	BCL-2–associated X protein (pro-apoptotic effector)
BCL-2	A proto-oncogene that promotes cell survival
BCRP	Breast cancer resistance protein
BI354 (IBI354)	Anti-HER2 antibody–drug conjugate (investigational code)
BRCA1/2	Breast Cancer Gene 1/2
CAD	Caspase-activated DNase
CARD	Caspase recruitment domain
CES	Carboxylesterases
CI	Confidence interval
CKD-602	Belotecan
cMOAT	Canalicular multispecific organic anion transporter
CPT	Camptothecin
CPT-11	Irinotecan
CRISPR-Cas9	Clustered Regularly Interspaced Short Palindromic Repeats–Cas9
CYP3A4	Cytochrome P450 3A4
DCR	Disease control rate
DED	Death effector domain
DISC	Death-inducing signaling complex
DLS	Dynamic light scattering
DNA	Deoxyribonucleic acid
DoR	Duration of response
DS-8201	Trastuzumab deruxtecan (development code)
DX-8951f	Exatecan
DXd	Deruxtecan payload
ECOG-PS	Eastern Cooperative Oncology Group Performance Status
Endo G	Endonuclease G
EPR	Enhanced permeation and retention
ERK	Extracellular signal-regulated kinase
FADD	Fas-associated death domain
FasL	Fas ligand
FDA	Food and Drug Administration
FL118	Novel camptothecin derivative (research code)
FOLFIRI	Folinic acid, fluorouracil, and irinotecan
FOLFIRINOX	Fluorouracil, leucovorin, irinotecan, and oxaliplatin
FTIR	Fourier Transform Infrared Spectroscopy
G-CSF	Granulocyte colony-stimulating factor
GG211	Lurtotecan
GI	Gastrointestinal tract
GLOBOCAN	International Agency for Research on Cancer (Global Cancer Observatory)
HAP1	Near-haploid human cell line
HER2	Human Epidermal Growth Factor Receptor 2
HIF	Hypoxia-inducible factor
HR	Hazard ratio
ICAD	Inhibitor of caspase-activated DNase
IMMU-132 (SG)	Sacituzumab govitecan
ISG15	Interferon-stimulated gene 15
Log P	Partition coefficient
MDR1	Multidrug resistance protein 1
MEK	Mitogen-activated extracellular signal-regulated kinase
mOS	Median overall survival
mPFS	Median progression-free survival
MC38	Murine colon adenocarcinoma cell line
MRP1	Multidrug resistance-associated protein 1
MRP2	Multidrug resistance-associated protein 2
mTOR mTTP	Mechanistic Target of Rapamycin Median Time To Progression
Nal-IRI	Nanoliposomal irinotecan
NEs	Nanoemulsions
NF-κB	Nuclear factor kappa-light-chain-enhancer of activated B cells
Noxa	Phorbol-12-Myristate-13-Acetate-Induced Protein 1 (PMAIP1)
NPs	Nanoparticles
NSCLC	Non-small cell lung cancer
OH	Hydroxyl
OPN	Osteopontin
ORR	Overall/Objective response rate
O/W	Oil-in-water (emulsion)
p53	Tumor suppressor protein
PFS	Progression-free survival
P-gp	P-glycoprotein
PK-tox	Pharmacokinetics–toxicity correlation
PNPs	Polymeric nanoparticles
PP	Progression parameters
RAD51	RAD51 recombinase
RAIDD	Receptor-interacting protein with death domain
RFS 2000	Rubitecan
RNA	Ribonucleic acid
RNAi	RNA interference
SCLC	Small-cell lung cancer
SEM	Scanning electron microscopy
SG	Sacituzumab govitecan
SII	Systemic immune-inflammatory index
Smac	Second mitochondrial caspase activator
SN-38	7-ethyl-10-hydroxycamptothecin
SNEDDS	Self-nanoemulsifying drug delivery systems
ST1481	Gimatecan
ST-1968	Namitecan
tBid	Truncated Bid
T-DXd	Trastuzumab deruxtecan
TEM	Transmission electron microscopy
TKI	Tyrosine kinase inhibitors
TNBC	Triple-negative breast cancer
TNF	Tumor necrosis factor
TNFR1	Tumor necrosis factor receptor 1
TNFR2	Tumor necrosis factor receptor 2
Topo I	Topoisomerase I
TPT	Topotecan
TRADD	Tumor necrosis factor receptor type 1-associated death domain protein
TRAIL	TNF-related apoptosis-inducing ligand
TROP-2	Trophoblast Cell Surface Antigen 2
UGT1A1	UDP-glucuronosyltransferase 1A1
VDAC	Voltage-dependent anion channel
WHO	World Health Organization
Wnt	Wingless-related integration site pathway
